# Metabolite profiling of bioactive compounds in tempe flour and its potential as a hypocholesterolemic functional food

**DOI:** 10.3389/fnut.2025.1622952

**Published:** 2025-11-12

**Authors:** Made Astawan, Anisha Ayuning Tryas, Saraswati Saraswati, Tutik Wresdiyati, Diana Nur Afifah, Frima Elda, Rifqi Ahmad Riyanto, Sastia Prama Putri

**Affiliations:** 1 Department of Food Science and Technology, Faculty of Agricultural Engineering and Technology, IPB University, Bogor, Indonesia; 2 School of Veterinary Medicine and Biomedicine Sciences, IPB University, Bogor, Indonesia; 3 Department of Nutrition Science, Faculty of Medicine, Universitas Diponegoro, Semarang, Central Java, Indonesia; 4 Nutrition Department, Faculty of Public Health, Universitas Andalas, Padang, Indonesia; 5 Bioresource Engineering Laboratory, Division of Advanced Science and Biotechnology, Fukusaki Laboratory Department of Biotechnology, Graduate School of Engineering, Osaka University, Suita, Japan; 6 Department of Food Technology, Faculty of Agriculture, Universitas Sultan Ageng Tirtayasa, Serang, Banten, Indonesia; 7 Department of Biotechnology, Graduate School of Engineering, Osaka University, Suita, Japan

**Keywords:** anti-lipase, GC–MS, cholesterol, metabolomic, tempe

## Abstract

High cholesterol is one of the risk factors for cardiovascular disease, which is currently a global health problem. This study aimed to compare the potential of tempe flour and soybean flour as functional foods for lowering blood lipid levels. This study evaluated both flour’s cholesterol-binding activity and anti-lipase activity in vitro. In addition, metabolomic profiling using GC-MS was conducted to identify bioactive compounds contributing to the hypolipidemic effect. The results showed that compared to soybean flour, tempe flour had a higher cholesterol-binding activity (27.66 vs. 14.98%, *p* < 0.05) and stronger anti-lipase activity (23.24 vs. 15.03%, *p* < 0.05). GC-MS analysis revealed that tempe flour was rich in isoflavones, amino acids, organic acids, meglutol, and GABA. These components are suspected to contribute to the hypolipidemic effect through mechanisms of inhibiting cholesterol synthesis, forming cholesterol esters, and increasing the excretion of cholesterol and bile acids. These findings suggest that tempe flour has greater potential as a functional food for preventing and managing dyslipidemia.

## 1 Introduction

Metabolic syndrome (MS) is a condition where the body is at high risk of developing chronic diseases such as cardiovascular disease (CVD). An individual is diagnosed with metabolic syndrome if they have at least three of the following six health conditions simultaneously: high blood glucose, high blood pressure, high blood fats or triglycerides, high blood cholesterol, low HDL cholesterol, and excess waist circumference/obesity (1). Elevated total cholesterol levels beyond the normal range in the blood are the most common health problems. Recently, this disease has not only been suffered by the elderly but also by the productive age group (2). The significant global burden of degenerative diseases underscores the urgent need for effective prevention strategies through the consumption of functional foods (3–5).

According to data from the Global Burden of Disease (GBD), in 2019, 3.78 million people worldwide died from ischemic heart disease, a condition associated with high LDL cholesterol levels in patients (6). GBD data also explains that countries in the Asian region have the highest number of deaths from ischemic heart disease and ischemic stroke due to high LDL cholesterol levels (7). In 2019, Indonesia was one of the Asian countries ranking fifth in the world with the highest number of deaths from heart disease (6). A healthy lifestyle, encompassing regular physical activity and a balanced, nutritious diet, is crucial for maintaining optimal cholesterol levels.

Indonesia produces a traditional fermented food derived from soybeans, shown in numerous studies to possess hypolipidemic and hypocholesterolemic properties. This food comes from the Central Java region, Indonesia, and is called tempe. Tempe is a multi-fermented product. The fermentation process of soybeans will increase their nutritional quality. The composition of amino acids, lipids, the antioxidant content of isoflavones, and carbohydrates will change into simpler monomers due to the hydrolysis process carried out by microorganisms (8–10). In addition, the fat content in tempe is also lower than in unfermented soybeans due to the hydrolysis of lipids into free fatty acids (11, 12). These components will be used by the fungus *Rhizopus* spp. as an energy source so that the fat content will decrease during the fermentation process. Based on the findings of Yuliani et al. (13) the tempe fermentation process can reduce the total fat content from 9.77%–13.59% in soybean seeds to 3.34%–5.37% in the final fermented tempe. Similarly, in a study by Polanowska et al. (14) tempe fermentation of faba bean samples also reduced total fatty acids by 38%–78%. The variation in reduction may be attributed to differences in lipase activity among tempe samples. Moreover, bioactive components from tempe fermentation, such as aglycone isoflavones, are more active and effective than soybeans in controlling blood cholesterol and triglyceride levels. Therefore, tempe is often referred to as a food for preventing obesity (15, 16).

Traditional Indonesian cuisine commonly features deep-fried tempe as a staple side dish, often served alongside rice. This common cooking method, however, may diminish the cholesterol-lowering benefits of tempe by introducing additional dietary fat. As reported by Yunigrum et al. (17) deep-frying increases the fat content of tempe, which can potentially elevate blood cholesterol levels. Alternative processing methods are necessary to fully leverage the cholesterol-lowering potential of tempe. Tempe flour and its derivatives offer a promising alternative for individuals with high cholesterol, as they retain significant nutritional value and may exhibit even greater cholesterol-lowering effects compared to soybean flour, as demonstrated by *in vivo* studies (18, 19). Tempe’s antioxidant compounds, which are more superior than those in unfermented soybeans, play a major role in preventing metabolic diseases. These antioxidants can work at the cellular level, especially in their extracted form (20). However, no exploratory research, such as a comparative metabolomic analysis to identify hypocholesterolemic compounds, or experimental studies, like those identifying the cholesterol-reducing ability of these two flours, have been conducted. Therefore, this study aims to fill these gaps by elucidating the underlying mechanisms by which tempe flour exerts its hypocholesterolemic effects. This will be achieved by evaluating the antioxidant activity (DPPH), isoflavone content, cholesterol-binding activity, and anti-lipase activity of both flours. Furthermore, metabolomic profiling using GC–MS will be employed to identify specific metabolites that may contribute to the observed bioactivities. By correlating these bioactivities with the metabolomic data, this research will provide a strong scientific foundation for the development of tempe flour as a functional food.

## 2 Materials and methods

### 2.1 Materials

The primary materials used in this study were soybeans and tempe, sourced from a small-scale enterprise (SME) in Bogor, Indonesia. The tempe produced by this SME has obtained Indonesia National Standard (SNI), HACCP, BPOM (Indonesia Food and Drug Surveillance Agency), and Halal certifications, indicating compliance with national and international food safety standards. The tempe production process at this SME adheres to good manufacturing practices (GMP) and meets export standards. The process involves cleaning soybean seeds, soaking and boiling, dehulling, inoculation with *Rhizopus* spp., plastic packaging, and fermentation at room temperature (28–30 °C) for 48 h.

### 2.2 Tempe and soybean flour production process

Tempe flour and soybean flour were processed separately according to the method described by Mahdi et al. (21). Fresh tempe was cut into 2 × 2 cm^2^ cubes and then comminuted using a chopper. Soybean flour was produced through the following steps: cleaning, soaking, boiling, dehulling, and comminuting using a chopper. Both the comminuted tempe and soybeans were dried using a fluidized bed dryer (FBD) at 50 °C for 6 h. Drying with a FBD at temperatures below 60 °C prevents the oxidation and degradation of antioxidant components like polyphenols (Elgamal et al.). Additionally, FBD ensures the sample’s surface dries evenly, a notable advantage over traditional tray or vacuum drying methods. Subsequently, the dried materials were ground into a fine powder using a grinder and sieved through a 100-mesh sieve.

### 2.3 Analysis of antioxidant activity

The antioxidant activity of the samples was determined using the 2,2-diphenyl-1-picrylhydrazyl (DPPH) radical scavenging assay. This method assesses the ability of antioxidant compounds in the samples to stabilize the DPPH radical. The procedure was adapted from Abdurrasyid et al. (22). Initially, 1 g of the powdered beverage sample was mixed with 10 mL of a methanol–water solution (80:20, v/v). The mixture was homogenized using a vortex mixer for 1 min and then centrifuged at 4 °C and 3,000 rpm for 45 min. Subsequently, 0.2 mL of the supernatant was added to 3.8 mL of 0.1 mM DPPH solution. The mixture was incubated in the dark for 30 min. The absorbance of the incubated samples was measured at 517 nm (A1) using a spectrophotometer. In parallel, a standard curve was constructed using serial dilutions of ascorbic acid (25–200 μg/mL) as a reference standard. This curve was used to convert the absorbance values into antioxidant capacity expressed as mg ascorbic acid equivalents (AAE)/100 g. Additionally, a blank sample (A0) was measured without the sample in the test solution. The DPPH radical scavenging activity was calculated using the following formula:


$$\%\text{Antioxidant activity}=\frac{A0-A1}{A0}\;x\;100\%.$$

### 2.4 Analysis of isoflavone content

The isoflavone content of tempe flour and soybean flour was determined using a high-performance liquid chromatography (HPLC) method as described by Astawan et al. (23). Two grams of each sample were mixed with 30 mL of a 1:4 hydrochloric acid: acetonitrile solution to hydrolyze isoflavone glycosides into isoflavone aglycones. The mixture then extracted using a sonicator for 30 min. Thenincubated in a water bath for 1 h. After the hydrolysis process was complete, the sample was cooled and centrifuged. The supernatant was collected and filtered using a 0.45 μm syringe filter. A 20 μL aliquot of the filtrate was injected into an HPLC system equipped with a C-18 column (15 cm × 4.6 mm i.d., 5 μm particle size). The separation was performed using a reverse-phase mode, elution isocratic with a mobile phase consisting of a methanol:1 mM ammonium acetate mixture (6:4, v/v) at a flow rate of 1 mL/min. The UV detector was set at a wavelength of 265 nm, column temperature: 30 °C. Subsequently, the isoflavones were quantified using external standards of daidzein and genistein and a calibration curve. The concentrations of daidzein and genistein in the samples were then calculated based on the sample peak areas and the calibration equations.

### 2.5 Analysis of cholesterol binding activity

Cholesterol binding activity was determined using the Lieberman–Burchard (LB) method, as described by Muharni et al. (24) and Imtihani et al. (25). The LB method is a colorimetric assay that reacts cholesterol with the LB reagent. Samples were extracted by maceration with 70% ethanol for 3 × 24 h at room temperature, followed by sonication for 30 min. Preliminary analysis indicated that the optimal wavelength for measurement was 415 nm with an operating time of 45 min. A standard curve was constructed using serial concentrations of cholesterol (100, 150, 200, 250, and 300 ppm) to quantify the cholesterol content. Five milliliters of each sample extract and simvastatin (positive control) were mixed with 300 ppm cholesterol solution (A) in test tubes, vortexed for 30 s, and incubated at 37 °C for 60 min. After incubation, the tubes were centrifuged at 4,000 rpm for 5 min. The supernatant was transferred to new tubes, 1 mL of acetic anhydride was added, and the mixture was vortexed for 30 s and kept in the dark for 30 min. Subsequently, 0.1 mL of concentrated sulfuric acid was added, vortexed, and incubated in the dark for 45 min. Finally, the absorbance of the solution was measured at 415 nm. The cholesterol concentration in the test samples and positive control (B) was determined using the standard curve equation. A blank sample (C) was also measured, which consisted of the sample solution without sulfuric acid, to serve as a color baseline. The calculation was performed using the following equation:


$$\%\text{Cholesterol binding activity}=\frac{A-(B-C)}{A}\times 100\%.$$

### 2.6 Analysis of anti-lipase

Pancreatic lipase inhibitory activity was determined according to the methods described by Pradono et al. (26) and Aji et al. (27). The assay was performed by mixing 0.1 mL of lipase (1 mg/mL) with 0.2 mL of 70% ethanol extract of the sample (the same for each concentration) and 0.7 mL of Tris–HCl buffer (pH 8.0) in a test tube. The mixture was homogenized and incubated at 37 °C for 15 min. After incubation, 0.1 mL of p-nitrophenyl palmitate (p-NPP) was added, and the mixture was incubated for an additional 30 min at the same temperature. Finally, the absorbance was measured at 410 nm using a spectrophotometer. Orlistat was used as a positive control (100% inhibition) and was prepared following the same procedure. The percentage of inhibition was calculated as follows:


$$\%\text{Inhibition}=\frac{A0-A}{A}\times 100\%$$

Note: A0, Absorbance of blank (without inhibitor); A, Absorbance of sample.

### 2.7 GC–MS analysis

#### 2.7.1 Sample extraction and derivatization

GC–MS analysis was preceded by sample extraction and derivatization. The extraction technique involved freezing and homogenization of the sample using a multi-beads shocker (Yasui Kikai, Osaka, Japan), as described by Prativi et al. (28). A 50 μg/mL solution of ribitol was added to each homogenized sample as an internal standard. At the end of the extraction process, 200 μL of the aqueous phase was obtained. A quality control (QC) sample was prepared by pooling 200 μL of the aqueous phase from all samples. Both the samples and QC were centrifuged using a centrifugal concentrator (Taitec Co., Tokyo, Japan) for 2 h at room temperature. In the second step, the extracted samples were derivatized by mixing the dried extract with 100 μL of methoxyamine hydrochloride and incubating at 30 °C for 90 min with stirring at 1200 rpm. Subsequently, 50 μL of N-methyl-N-trimethylsilyltrifluoroacetamide (MSTFA) was added to the sample, followed by incubation at 37 °C for 30 min with stirring at 1200 rpm (Eppendorf Ltd.). The resulting samples were transferred to GC vials for GC–MS analysis.

#### 2.7.2 GC–MS sample injection

The derivatized samples were immediately injected into the GC–MS instrument using an AOC-20i/s autosampler (Shimadzu) in split mode (25:1, v/v) at 230 °C. A 30 × 0.25 mm i.d. fused silica capillary column coated with 0.25 μm InertCap 5MS/NP (GL Science, Inc.) was used. Hydrogen was used as the carrier gas at a flow rate of 1.12 mL/min and a linear velocity of 39 cm/s. The initial column temperature was 80 °C for 2 min, followed by an increase to 330 °C at a rate of 15 °C/min and held for 6 min. Electron ionization (EI) was used as the ionization method, with an ion source temperature of 200 °C, and the mass-to-charge ratio (m/z) range was set from 85 to 500 with a scan rate of 6.67 scans per second. Retention indices (RI) were determined using a standard alkane mixture. In the final step, the data from the GC–MS instrument, in the form of chromatograms, was converted to netCDF format using the GC–MS-QP2010 Ultra instrument software (Shimadzu, Kyoto, Japan). The data was then exported to a CSV file. Retention indices (RI) were calculated based on the retention times of the standard alkane mixture. The RI and mass spectra were compared to the database in the instrument to identify the compounds in the samples.

### 2.8 Statistical data analysis

The antioxidant activity data was analyzed using SPSS 22. An independent *t*-test was performed at the 5% significance level. For multivariate data from GC–MS analysis, visualization was performed using SIMCA 18 (Umetrics, Umea, Sweden). Principal component analysis (PCA) was initially applied to the multivariate data to visualize sample discrimination and assess the initial model quality. If the PCA was satisfactory, orthogonal projection to latent structures (OPLS) analysis was performed. OPLS analysis generated a correlation model between metabolites (X) and bioactivity (Y). The output data from OPLS analysis included score plots, S-plots, Y-related coefficient plots, and VIP plots. The generated model was validated using R^2^X, R^2^Y, Q^2^, CV-ANOVA, and permutation tests.

## 3 Results and discussion

### 3.1 Antioxidant activity and isoflavone content of tempe flour and soybean flour

Soybeans and tempe contain a variety of antioxidant compounds. Consuming foods rich in antioxidants offers numerous health benefits. Many chronic diseases and metabolic syndromes are caused by oxidative stress in the body (29). The isoflavones in soybeans and tempe can prevent this from happening. Soybean isoflavones can act as anti-atherosclerotic agents by inhibiting oxidative stress, increasing nitric oxide (NO) availability, reducing LDL size, and preventing LDL peroxidation (30). Table 1 shows that the antioxidant activity of tempe flour (31.56 ± 2.27%) was significantly higher (*p* < 0.05) than that of soybean flour (27.99 ± 1.57%). This result is consistent with the isoflavone content of the samples. The content of daidzein and genistein in tempe flour was also significantly higher (*p* < 0.05) compared to soybean flour.

**Table 1:** Antioxidant profile of soybean flour and tempe flour.

Antioxidant profile	Soy flour	Tempe flour
DPPH radical scavenging activity (%)	27.99 ± 1.57^a^	31.56 ± 2.27^b^
Antioxidant capacity (mg AEAC/100 g)	33.75 ± 0.02^a^	38.00 ± 0.03^b^
Daidzein (μg/g)	213.14 ± 0.15^a^	840.20 ± 0.10^b^
Genistein (μg/g)	398.32 ± 1.19^a^	1577.52 ± 5.13^b^

Values in a row followed by different letters indicate significant differences (*p* < 0.05).

Based on Barus et al.’s study (31), the antioxidant activity of tempe can reach 53%–84%. The values might decrease in powdered samples due to the drying process, which can reduce bioactive antioxidant components. However, this finding is consistent with the study by Kuligowski et al. (32) who observed that the isoflavones daidzein and genistein increased by 5–8 times during tempe fermentation. This demonstrates that the content of both isoflavones is indeed higher in tempe than in soybeans. The fermentation process in tempe increases the aglycone forms of daidzein and genistein due to the activity of microorganisms that hydrolyze the glycosidic bonds, namely β-glucosidase (23). The increase in aglycone isoflavones enhances their bioavailability and antioxidant activity, as both compounds are flavonoids that can donate electrons to free radicals. In addition to isoflavones, which are derivatives of flavonoids, soybeans also contain other antioxidant compounds such as phenolic compounds.

### 3.2 Cholesterol binding activity

**Figure 1:**
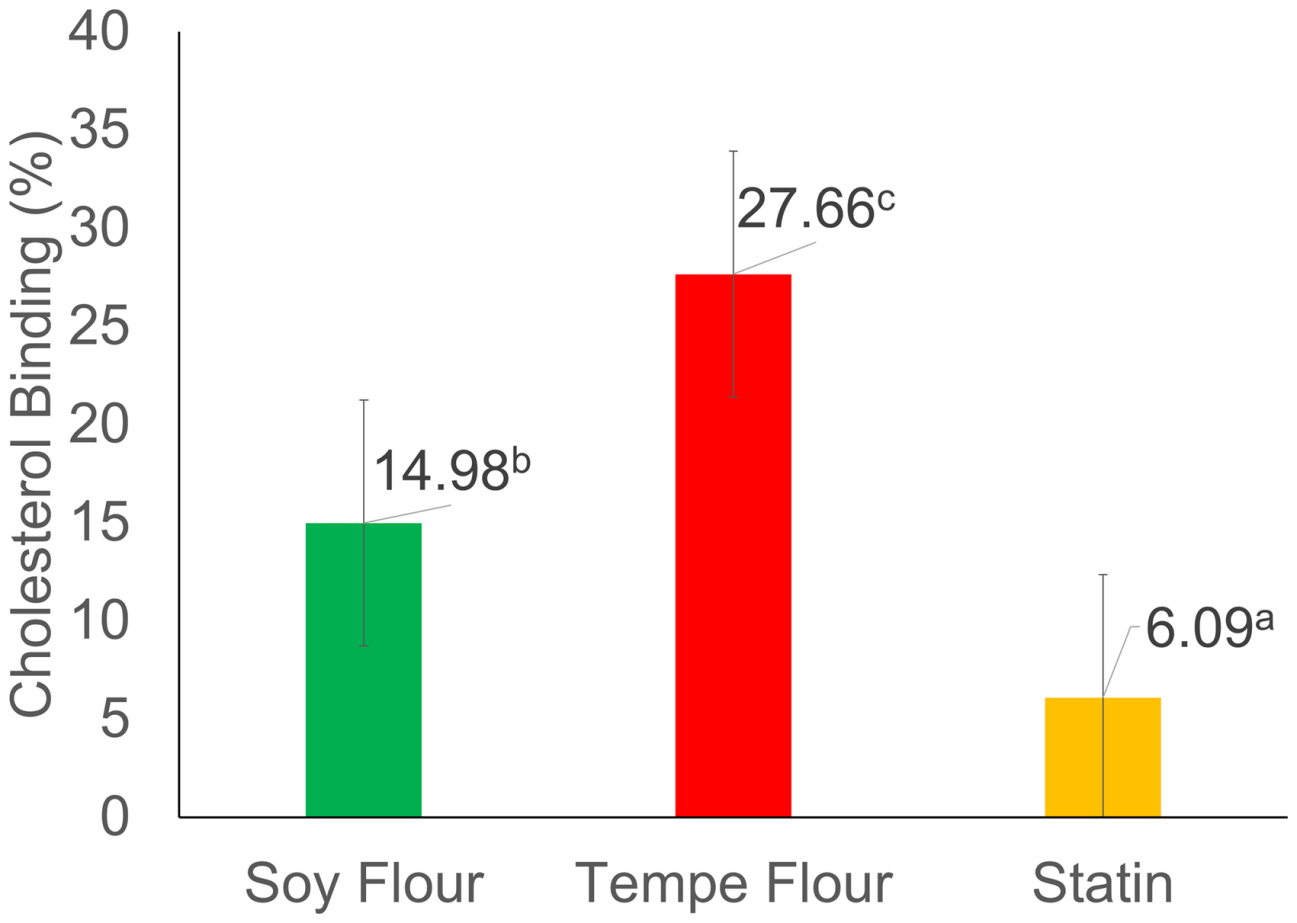
Cholesterol binding activity. Values followed by different letters indicate significant differences (*p* < 0.05).

The results of the Lieberman–Burchard analysis of tempe flour and soybean flour (Figure 1) showed significantly different binding percentages. Tempe flour had the highest binding value (27.66%), followed by soybean flour (14.98%), and the lowest was the positive control, statin (6.09%). These results indicate that tempe flour and soybean flour have a greater potential to control blood cholesterol levels compared to statins, which work through a different mechanism. The positive control, statin, showed the lowest percentage decrease in cholesterol, presumably due to the lack of direct binding with free cholesterol. This limited cholesterol-lowering effect of statins can be attributed to their mechanism of action, which primarily involves inhibiting HMG-CoA reductase, the rate-limiting enzyme in cholesterol biosynthesis, rather than directly binding to cholesterol molecules (33). The findings are largely comparable to the study by Zendrato et al. (34), where Porang glucomannan extract exhibited a cholesterol-binding capacity of 13.42–45.56% (at concentrations of 150–750 μg/mL). In contrast, research by Musa et al. (35) on *Saurauia vulcani* Korth. (Actinidiaceae), a tropical medicinal plant, reported a higher cholesterol-binding capacity ranging from 20 to 80%.

The cholesterol-binding activity of tempe flour and soybean flour was analyzed using the photometric Lieberman–Burchard (LB) method. The LB method can be used for both qualitative and quantitative analysis. The principle of this analysis involves measuring the amount of free cholesterol in a solution that is not bound to metabolites in the sample. Free cholesterol undergoes derivatization with acetic anhydride and H_2_SO_4_, resulting in a strongly chromogenic blue–green cholesterol derivative, known as an aromatic sulfonic acid. The unbound cholesterol was quantified spectrophotometrically using a method adapted from Adu et al. (36). A higher absorbance value correlates with a higher concentration of free cholesterol, as evidenced by a more intense color of the solution. Conversely, if a sample effectively binds cholesterol, the resulting solution will exhibit a lighter color and a lower absorbance value. The samples used in the cholesterol binding activity test were ethanol extracts. Ethanol was chosen as the extraction solvent because it is considered a safe solvent and can effectively extract bioactive compounds, especially antioxidant derivatives such as phenolics, flavonoids, and saponins from the material. Ethanol can degrade non-polar walls through semi-polar interactions, allowing intracellular phenolic-flavonoid compounds to be extracted (37–39).

A positive correlation was also found between the cholesterol-binding activity and the antioxidant activity of the samples; the higher the antioxidant activity, the greater binding cholesterol activity. The cholesterol binding mechanism in the LB reaction involves the interaction of the cholesterol hydroxyl (-OH) group with antioxidant compounds, such as phenols and flavonoids. This interaction leads to the formation of stable hydrogen bonds, effectively preventing cholesterol from reacting with sulfuric acid. The crucial role of the hydroxyl group in this process is underscored by its involvement in the formation of a pharmacophore, as described by Musa et al. (35). The OH group in phenols is also thought to have the potential to bind with cholesterol, forming hydroxysterols. Hydroxysterols are another form of cholesterol that has accepted an oxygen atom. The remaining free cholesterol reacts with acetic anhydride and H_2_SO_4_, forming a colored compound.

This model of cholesterol binding in the LB analysis represents one of the body’s regulatory mechanisms for maintaining stable cholesterol levels (homeostasis). Cholesterol homeostasis can be controlled by four mechanisms: (1) cholesterol absorption from food through the small intestine, (2) cholesterol synthesis by various body tissues, especially the liver, and intestines, for release into the blood plasma, (3) cholesterol excretion through the excretory system regulated by the liver, and (4) conversion of cholesterol to various hormone compounds required by humans (40). Cholesterol exists in two forms: free cholesterol and cholesterol ester. Only monomeric cholesterol can be absorbed by the small intestine/basolateral membrane (41). Thus, bound cholesterol is presumably unable to be absorbed or is inhibited from absorption and can be excreted through feces, similar to the mechanism of dietary fiber binding bile acids or cholesterol. The percentage of cholesterol binding in this LB analysis is expected to reflect the difference in the cholesterol binding capacity of the samples. Tempe flour and soybean flour are known to have the potential to control blood cholesterol levels.

#### 3.2.1 Lipase inhibition activity

**Figure 2:**
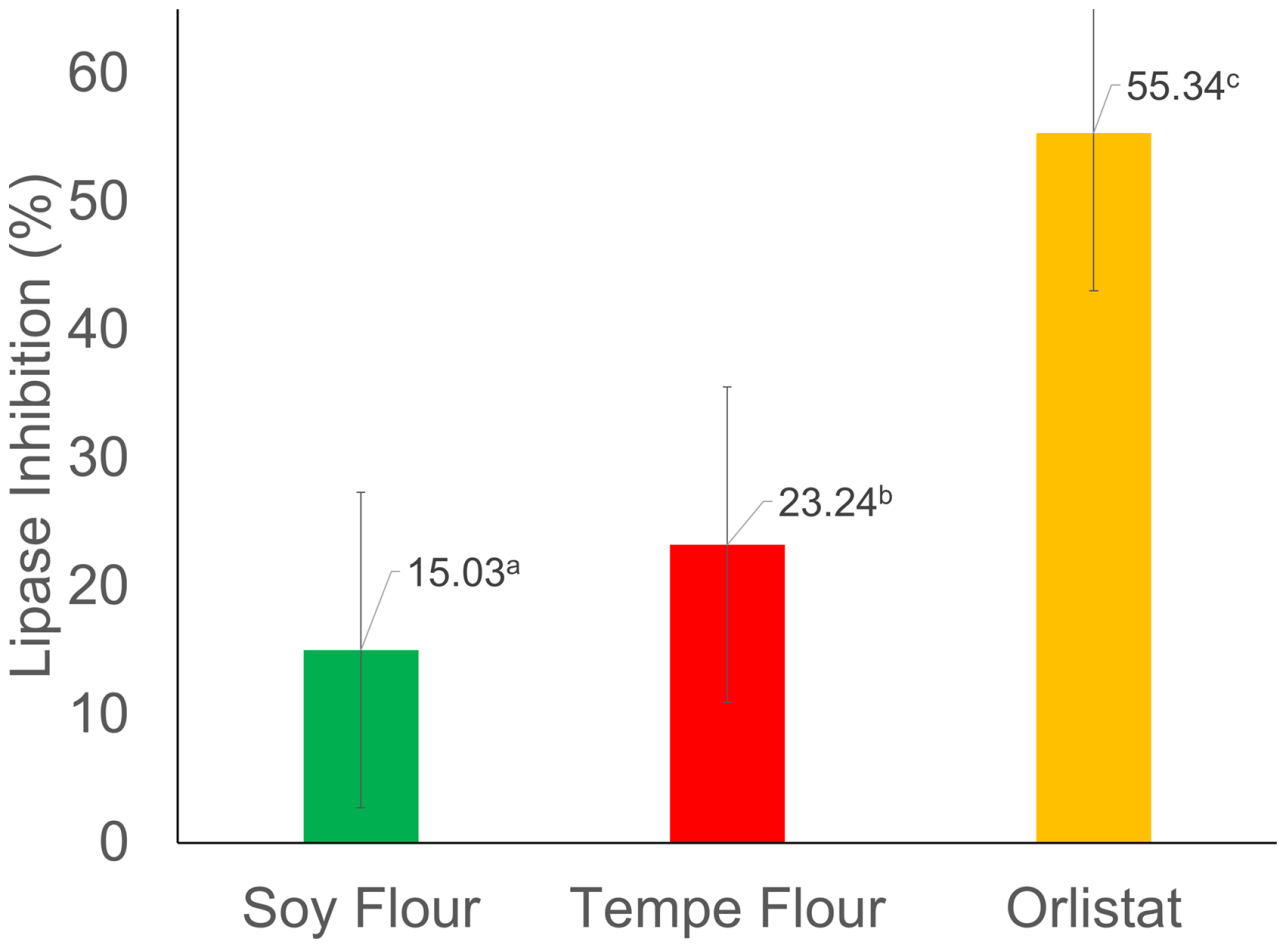
Percentage of lipase inhibition. Values followed by different letters indicate significant differences (*p* < 0.05).

Tempe flour extract had a higher inhibition value (23.24%) compared to soybean flour extract (15.03%), but both were much lower than the inhibition by the positive control, orlistat (55.34%). Figure 2 shows that tempe and soybean flour extracts had significantly different percentages of lipase inhibition. The lipase inhibition activity of the tempe sample in this study was higher than that of the cinnamon leaves sample (16.23%) in the research by Megawati et al. (42), yet lower than the activity found in the fruit of *Solanum stramonifolium* (33.4–94.6%), a plant native to Thailand (43). Meanwhile, the positive control, Orlistat, in this study had a value nearly identical to the one reported by Megawati et al. (42), who stated that orlistat has a high inhibition value of 46.79%. Orlistat, an anti-obesity medication, exerts its therapeutic effect by irreversibly inhibiting pancreatic lipase, a key enzyme involved in the hydrolysis of triglycerides, at its active site (44). Therefore, it has the highest inhibition value. The inhibition mechanism in tempe and soybean flour extracts is suspected to be due to the presence of proteins, peptides, and antioxidants (15).

Lipase, a critical enzyme in lipid metabolism, is a hydrolase that catalyzes the hydrolysis of dietary triglycerides into fatty acids and glycerol within the small intestine. The anti-lipase analysis aims to assess the ability of compounds to inhibit lipase activity, thereby preventing the digestion and subsequent absorption of dietary lipids. Consequently, these components cannot be absorbed by the small intestine and are excreted in the feces. If a compound has this ability, it has the potential to be a candidate for obesity prevention (45).

*In vitro*, the anti-lipase activity of tempe flour and soybean flour extracts was assessed using a colorimetric spectrophotometric assay. This method simulates the physiological conditions of the human digestive system, requiring pancreatic lipase as the primary enzyme along with its substrate and necessary cofactors to facilitate the enzymatic reaction (27). Pancreatic lipase, known for its high hydrolysis efficiency (50%–70%) for dietary lipids, was employed as the enzyme source in this study (46).

*In vitro*, anti-lipase activity analysis in soybeans is influenced by the content of antioxidant compounds, such as phenols and saponins, which can inhibit the enzyme’s ability to hydrolyze lipid substrates (47). In this study, it was found that tempe flour had higher antioxidant activity and isoflavone content compared to soybean flour. Phenolic compounds, including flavonoids, inhibit lipase activity by forming stable, irreversible bonds within the enzyme’s active site. The inhibitory potency of these compounds is influenced by their structural complexity, with more complex structures generally exhibiting greater inhibitory activity (48, 49). Polyphenols and saponins inhibit lipase through a competitive mechanism, competing with the substrate at the active site of the enzyme reversibly (50).

The differences in the percentage of lipase enzyme inhibition can be influenced by the extraction method, the type of solvent used, and the solvent-to-sample ratio in the extraction, which affect the bioactive components that can be extracted from the test sample (51). The ability to strongly inhibit lipase enzymes can affect blood cholesterol levels. One mechanism is that when the body breaks down fewer triglycerides, the transport of triglycerides from the lumen to the liver via the bloodstream, which requires a chylomicron structure (lipoprotein), decreases. When there are no triglycerides to transport, chylomicrons are not formed, and thus the amount of cholesterol in the blood also decreases (52).

### 3.3 Bioactive compound profile and its correlation with bioactivity

The results showed that 58 metabolites were detected (Table 2). Of these, 52 compounds were successfully annotated using an in-house library, and only six compounds remained unidentified. Of the 52 annotated compounds, there were six bioactive compounds in soybean flour and tempe flour, namely 3-hydroxy-3-methylglutaric acid, 4-aminobutyric acid, meso-erythritol, 3-phenyllactic acid, daidzein, and genistein. In addition, there were 20 amino acids and their derivatives, 8 organic acids and their derivatives, 10 carbohydrates and their derivatives, 1 mineral, and 7 other compounds.

**Table 2:** Annotated metabolite by GC–MS/MS.

Annotated metabolite	
*Amino acids and their derivatives*	*Organic acids and their derivatives*
Alanine	Malonic acid
Aspartic acid	Saccharic acid
Glutamic acid	Fumaric acid
Pyroglutamic acid	Lactic acid
Asparagine	Malate acid
Phenylalanine	Citric acid
Glycine	Succinic acid
Glutamine	
Histidine	*Sugar and their derivatives*
Isoleucine	Galactose
Leucine	Glucose
Lysine	Lactitol
Ornithine	Mannitol
Proline	Raffinose
Serine	Sucrose
Threonine	Trehalose
Thymine	Xylitol
Tyrosine	Sorbitol
Tryptophan	Melibiose
Valine	
	*Bioactive compound*
*Mineral*	3-Hydroxy-3-Methylglutaric acid
Phosphate	4-Aminobutyric acid
	Daidzein
*Others*	Genistein
2-Aminoethanol	3-Phenyllactic acid
Glycerol	Meso-erythritol
Inositol	
N-acetyl-alpha-D-glucosamine 1-phosphate	
Pyrogallol	
Urasil	
Xanthine	
Urea	

**Figure 3:**
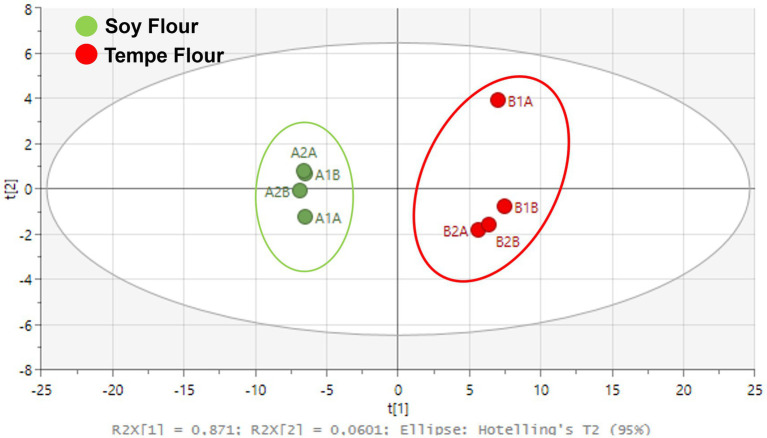
Principal component analysis (PCA) score plot of the chemical profiles of tempe flour and soybean flour.

The GC–MS metabolomic data was processed using SIMCA 18 and showed significant differences in the characteristics of soybean flour and tempe flour based on their metabolites. PCA analysis showed that both samples could be well clustered in different quadrants (Figure 3). The metabolite components in tempe flour clustered in the positive quadrant relative to the x-axis, while the metabolite components in soybean flour were separated and located in the negative quadrant relative to the x-axis. Both flours had distinct compounds.

Multivariate data analysis using PCA can illustrate class differences in a dataset, such as mapping differences in chemical profiles (53). The output of PCA is a score plot. The R^2^X value obtained from this PCA model was 0.87 with a Q^2^ value of 0.85. Both values are greater than 0.4, indicating that the resulting model is a good representation of the data (54). Metabolomics analysis serves as a valuable approach for comprehensively characterizing the dynamic metabolic shifts that occur during fermentation, enabling a deeper understanding of the biochemical alterations within the food matrix. Fermentation in food alters the food structure, which can increase or decrease the levels of certain compounds due to degradation processes (55). In the field of foodomics, metabolomics analysis is useful for comprehensively capturing these changes at one time. The complexity of fermentation products can be unraveled by identifying thousands of metabolites using analytical instruments. The metabolomics method used was an untargeted profiling analysis or an exploratory method that does not limit the discovery of specific compounds. By employing an untargeted approach, this study maximizes the potential for identifying a diverse range of metabolites, including novel compounds with potential biological significance (56).

**Figure 4:**
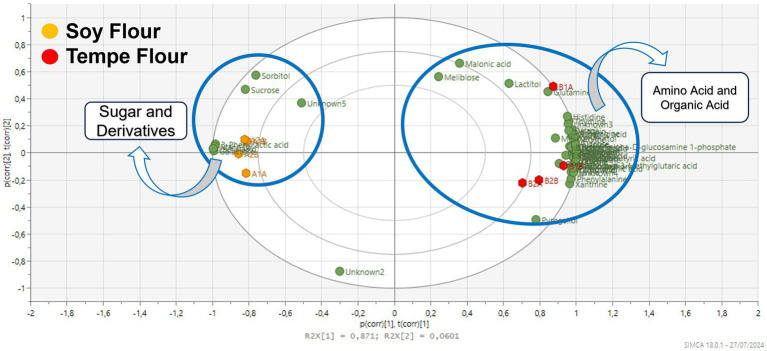
Loading plot of the chemical profile of tempe flour and soybean flour.

Tempe fermentation is the result of synergistic activity between bacteria and fungi. Lactic acid bacterial fermentation is the first stage of the tempe-making process, which occurs during the soaking of boiled soybeans for 12 h at room temperature. Lactic acid bacteria utilize available sugars within the soybean substrate for growth and metabolic activity, resulting in the production of organic acids. This microbial activity leads to a significant reduction in sugar content, as evidenced by the diminished levels observed in tempe flour (Figure 4).

Tempe fermentation, facilitated by the action of *Rhizopus* spp. fungi, results in the accumulation of amino acids due to the degradation of proteins. The proteolytic activity of *Rhizopus* spp. leads to an increase in amino acids such as lysine, leucine, valine, and tyrosine in tempe, contributing to its characteristic bitter taste (28). Fermentation significantly alters the metabolic composition of soybeans, influencing both nutritional and sensory characteristics (28).

Metabolomic analysis revealed the presence of several derivative amino acids, including ornithine and pyroglutamic acid. Ornithine, a non-protein amino acid derived from arginine, has been shown to exhibit stress-relieving and sleep-promoting effects in clinical studies (57), while pyroglutamic acid is a derivative of glutamic acid. Pyroglutamic acid, another non-protein amino acid, is commonly found in fermented products produced by lactic acid bacteria (58) and exhibits anti-inflammatory and antidepressant effects (59). Both ornithine and pyroglutamic acid were found in higher quantities in tempe flour compared to soybean flour.

### 3.4 OPLS-based classification of tempe flour and soy flour based on cholesterol binding and anti-lipase activities

**Figure 5:**
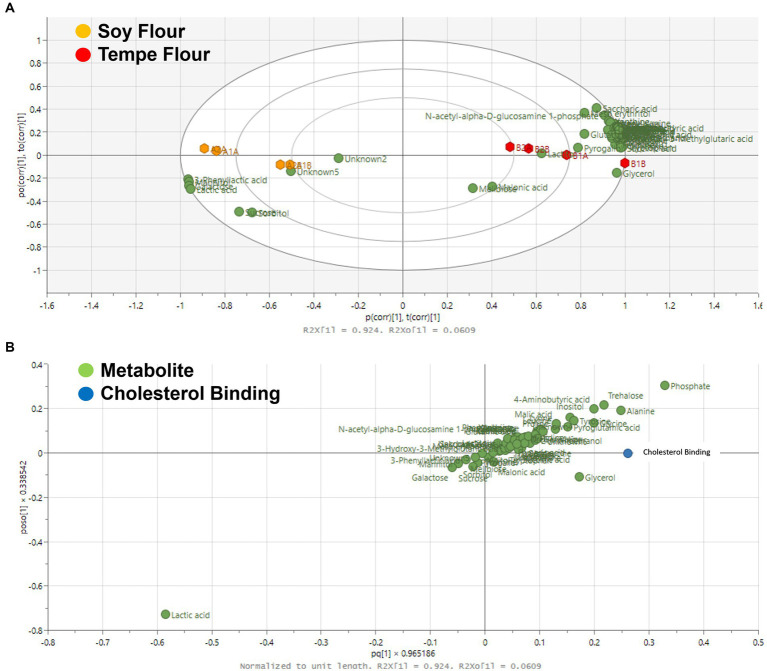
Score plot OPLS **(A)** and loading plot OPLS **(B)** based on cholesterol binding activity. **(A)** The x-axis (t[1]) and y-axis (t[2]) represent the predictive and orthogonal components, respectively. The model parameters R^2^X[1] = 0.924 and R^2^X[2] = 0.0609 indicate a strong predictive ability. The plot clearly shows that soy flour and tempe flour samples form two distinct clusters, indicating significant differences in their metabolite composition. **(B)** The x-axis (p[1]) and y-axis (p[2]) show the correlation of each metabolite with the predictive and orthogonal components. Cholesterol is highlighted as a significant marker distinguishing the two groups, confirming it is one of the key metabolites responsible for the separation between soy flour and tempe flour.

The OPLS score plot with Pareto scaling in Figure 5A illustrates the grouping based on cholesterol binding activity. Tempe and soybean flours are consistently separated into distinct quadrants. The OPLS model exhibited excellent performance with R^2^X = 0.98, R^2^Y = 0.99, and Q^2^ = 0.92, indicating a strong model fit. Figure 5B reveals that the cholesterol binding points align with the tempe flour samples in the positive quadrant of the x-axis. This suggests that the high cholesterol binding activity is likely attributed to the group of metabolites located in the same quadrant. Tempe flour samples clustered closely together in the same quadrant as the cholesterol binding points, indicating that tempe contains metabolites that significantly contribute to its high cholesterol binding activity. Metabolites close to the cholesterol points included amino acids and organic acids. These metabolite components are hypothesized to have a positive correlation with cholesterol-binding bioactivity.

Orthogonal Projections to Latent Structures (OPLS) analysis was employed to examine the correlation between cholesterol binding activity and anti-lipase activity with the metabolite profiles of the two samples. OPLS can be used to assess the relationship between two data matrices, such as chemical profiles and bioactivity data (60). Consequently, this analysis can identify metabolites that contribute to bioactivity. The output of OPLS includes score plots, S-plots, Y-related coefficient plots, and VIP plots. The resulting OPLS model was validated using permutation tests.

**Figure 6:**
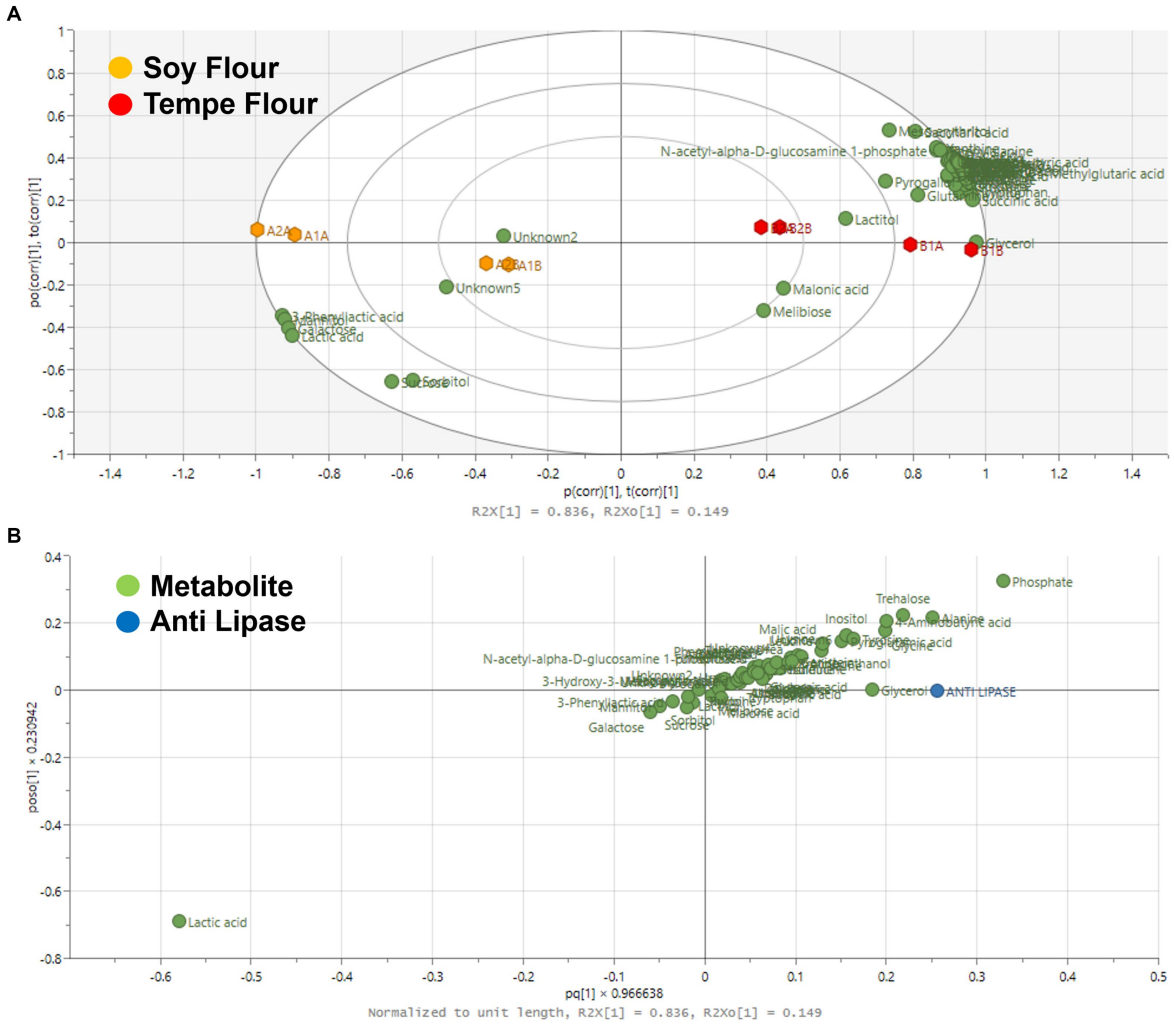
Score plot OPLS **(A)** and loading plot OPLS **(B)** based on lipase inhibition activity. **(A)** The x-axis (t[1]) and y-axis (t[2]) represent the predictive and orthogonal components, respectively. The model parameters R^2^X[1] = 0.836 and R^2^X[2] = 0.149 indicate a strong predictive ability. The plot clearly shows that soy flour and tempe flour samples form two distinct clusters, indicating significant differences in their metabolite composition. **(B)** The x-axis (p[1]) and y-axis (p[2]) show the correlation of each metabolite with the predictive and orthogonal components. Anti lipase is highlighted as a significant marker distinguishing the two groups, confirming it is one of the key metabolites responsible for the separation between soy flour and tempe flour.

Figure 6A presents the OPLS score plot, demonstrating the grouping of samples based on their lipase inhibition activity. Similar to the cholesterol-binding activity, tempe and soybean flour samples cluster distinctly in separate quadrants. The model exhibits excellent performance with R^2^X = 0.98, R^2^Y = 0.99, and Q^2^ = 0.93. Tempe samples consistently cluster in the positive quadrant, while soybean flour samples group in the negative quadrant relative to the x-axis.

The OPLS loading plot (Figure 6B) illustrates the relationship between individual metabolites and lipase inhibition. The lipase inhibition point is in the positive quadrant of the x-axis, closely aligned with one tempe sample but within the same quadrant as three other tempe samples. Similarly to the cholesterol-binding activity, all soybean flour samples are positioned in the opposite quadrant. Metabolites closest to the lipase inhibition point, such as amino acids, exhibit a positive correlation with lipase inhibition activity. This suggests that a higher abundance of these metabolites in tempe samples is associated with increased lipase inhibition.

### 3.5 Identification of key metabolites

To specifically identify the metabolites contributing to the OPLS model and their correlation with cholesterol binding and anti-lipase activities, Y-related coefficient, VIP, and S-plot analyses were conducted. Metabolites with VIP scores greater than 1 are considered to have a strong influence on the OPLS model and the separation of samples based on the relationship between X (metabolites) and Y (bioactivity) (61). Positive correlation coefficients (>0) indicate a strong positive correlation, while negative coefficients (<0) suggest a negative or reducing correlation (62). S-plots visualize the intensity of the correlation between metabolites and bioactivity based on their quadrant location. Metabolites in the upper right quadrant have a strong positive correlation, while those in the lower left quadrant have a weak negative correlation. Metabolites in the center have a negligible correlation. The combination of VIP, Y-related coefficient, and S-plot provides a comprehensive assessment of the importance of metabolites in the OPLS model and their relationship with bioactivity.

Supplementary Figures S1A–C, S2A–C present the scores for Y-related coefficient, S-plot, and VIP for cholesterol binding and lipase inhibition activities, respectively. Both bioactivities showed a strong positive correlation with amino acids and organic acids, as indicated by the Y-related coefficient and S-plot. Of the analyzed metabolites, 50 exhibited a positive correlation coefficient (>0), while 8 had a negative correlation coefficient (<0) in the Y-related coefficient plot.

The highest correlation score for both bioactivities was observed for phosphate metabolites, while the lowest score was found for lactic acid (−2.28). These results align with the S-plot, which shows phosphate metabolites in the upper right quadrant and lactic acid in the lower left quadrant. A significant advantage of the S-plot is its ability to visually identify metabolites with negligible correlations to bioactivity, as these metabolites are scattered in the central region of the plot. The study revealed that several organic acids, such as malonic acid and phenyl lactic acid, as well as certain carbohydrate derivatives, exhibited weak or negligible correlations with bioactivity. Metabolites with negligible correlations had correlation values close to zero.

To identify the specific metabolites contributing to model formation and sample separation, VIP scores were calculated for both models. The results showed that both models had 12 metabolites with VIP scores greater than 1.0, as presented in Table 3, and Y-related coefficient values ranging from 0.4 to 1.29. Lactic acid was the only metabolite that exhibited a negative correlation with both bioactivities but was a dominant contributor to the OPLS modeling. Of the remaining 11 biomarker metabolites, one was a mineral (phosphate), two were carbohydrates or their derivatives (trehalose and inositol), one was a lipid component (glycerol), four were amino acids (alanine, glycine, tyrosine, valine), and three were organic acids (4-aminobutyric acid, malic acid, pyroglutamic acid).

**Table 3:** Y-related coefficient and VIP score of bioactivity.

No.	Metabolite	Y-related coefficient score*	Metabolite	VIP score**
Cholesterol binding				
1	Phosphate	1.28	Lactic acid	4.64
2	Alanine	0.97	Phosphate	2.59
3	Trehalose	0.84	Alanine	1.94
4	Glycine	0.78	Trehalose	1.71
5	4-Aminobutyric acid	0.77	4-Aminobutyric acid	1.57
6	Glycerol	0.66	Glycine	1.55
7	Tyrosine	0.63	Glycerol	1.35
8	Inositol	0.60	Tyrosine	1.27
9	Pyroglutamic acid	0.58	Inositol	1.23
10	Malic acid	0.50	Pyroglutamic acid	1.19
11	Valine	0.50	Malic acid	1.03
12	Unknown6	0.41	Valine	1.00
Anti-lipase				
1	Phosphate	1.29	Lactic acid	4.62
2	Alanine	1.00	Phosphate	2.59
3	Trehalose	0.86	Alanine	1.96
4	4-Aminobutyric acid	0.78	Trehalose	1.71
5	Glycine	0.78	4-Aminobutyric acid	1.57
6	Glycerol	0.71	Glycine	1.55
7	Tyrosine	0.64	Glycerol	1.39
8	Inositol	0.61	Tyrosine	1.28
9	Pyroglutamic acid	0.58	Inositol	1.23
10	Valine	0.51	Pyroglutamic acid	1.18
11	Malic acid	0.51	Malic acid	1.02
12	Unknown6	0.42	Valine	1.01

^*^ The Y-related coefficient identifies metabolite compounds that have a strong or positive correlation with bioactivity. A score approaching one indicates the component with the strongest correlation.

^**^Variable importance in projection (VIP) is a measure used to assess the contribution of variables in the model to the explained variance. A higher VIP value indicates that a variable plays a more significant role in forming the OPLS model, which separates the tempe flour and soybean flour sample groups based on their bioactivity.

Phosphates, trehalose, inositol, and glycerol, metabolites formed during fermentation, contributed significantly to the clustering of tempe and soybean flour samples in PCA and OPLS analyses. The presence of these metabolites in higher concentrations in tempe flour can be attributed to the action of fungal enzymes. For instance, *Rhizopus* spp. possess phytase, which hydrolyzes phytate into phosphate and inositol (63), while yeasts metabolize glucose to produce trehalose and glycerol (8, 64, 65). Regarding the bioactivity of cholesterol binding and lipase inhibition, there have been no reports that the four metabolites identified can directly bind to cholesterol or inhibit lipase activity. Phosphate, for example, is an essential mineral for human health, particularly for bone health. However, a study by Trauttvetter et al. (66) found that the minerals calcium and phosphorus can form amorphous molecules that bind to bile acids in the small intestine, thus reducing blood cholesterol levels. Similarly, a meta-analysis by Tabrizi et al. (67) reported that inositol supplementation in patients with metabolic diseases can help improve blood lipid profiles by reducing cholesterol and triglyceride levels. Likewise, trehalose, a disaccharide component, can improve blood lipid profiles. *In vivo* studies have shown that trehalose can suppress fat absorption in the small intestine and increase its excretion via feces (68).

Amino acids and organic acids were also major contributors to the increased bioactivity and the OPLS modeling. While the specific mechanism of alanine’s influence on lipid metabolism remains unclear, glycine has been shown to reduce the risk of heart disease by lowering homocysteine levels (69). Branched-chain amino acids like valine have anti-atherogenic effects, and when combined with leucine, can reduce triglyceride and total cholesterol levels while increasing HDL-cholesterol (70). Furthermore, a synergistic effect between tyrosine and tryptophan has been observed, where specific ratios of these amino acids can effectively normalize triglyceride levels and reduce LDL-cholesterol in animal models of high-fat diet-induced obesity (71).

Gamma amino butyric acid (GABA) or 4-aminobutyric acid is similar compound which have four-carbon non-protein amino acid (72), a neurotransmitter, has been reported to have beneficial effects in preventing dyslipidemia. Malic acid and pyroglutamic acid, with VIP values of 1.02–1.19, indicated their significant roles in cholesterol binding and lipase inhibition. Studies on plums, rich in malic acid, have shown anti-lipase activity (73). Given that lipase activity is optimal at a pH of 7.5–8.5 (74), the presence of organic acids in fermented products may inhibit lipase activity by creating an acidic environment (75).

### 3.6 Model validation

The OPLS models were validated using R^2^Y and Q^2^Y values, with values closer to 1 indicating a better fit. The obtained R^2^Y and Q^2^Y values for both cholesterol binding and lipase inhibition models were high, suggesting good model performance (Supplementary Figure S3). Further validation was performed using permutation tests and CV-ANOVA (Supplementary Tables S1, S2), which confirmed the robustness of the models. While the study provided valuable insights, the relatively small sample size (*n* = 8) limited the generalizability of the findings. Future studies with larger sample sizes are recommended to enhance the robustness of the models and provide more conclusive evidence.

### 3.7 Bioactive components in tempe and soybean flours

**Figure 7:**
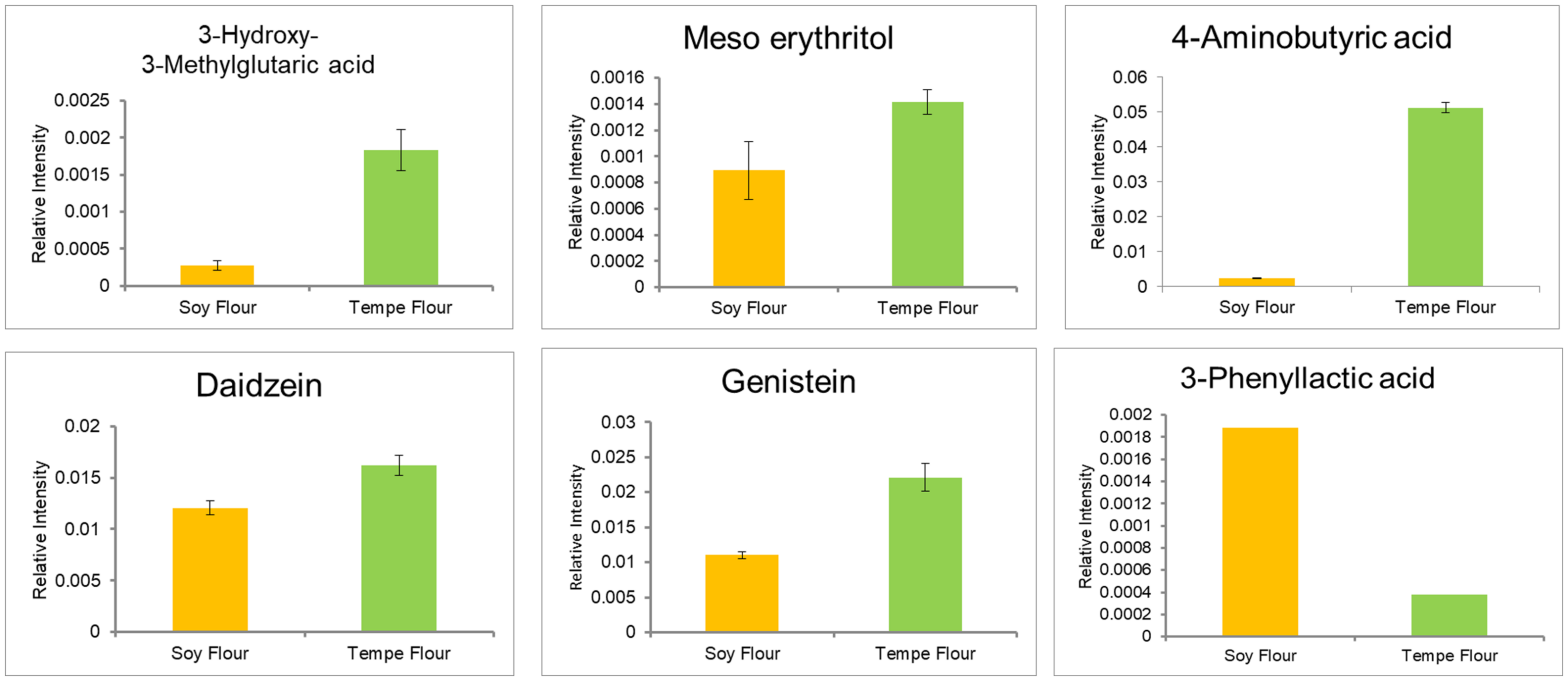
Bioactive compound of tempe flour and soybean flour.

GC–MS analysis identified six dominant bioactive compounds: 3-hydroxy-3-methylglutaric acid, 4-aminobutyric acid (GABA—Gamma aminobutyric acid), meso-erythritol, daidzein, and genistein in tempe flour, while 3-phenyllactic acid was predominantly found in soybean flour (Figure 7). These bioactive compounds are known to exert various effects on cholesterol metabolism. Based on the literature, only meso-erythritol and 3-phenyllactic acid have not been directly linked to cholesterol metabolism.

Meso-erythritol, a sugar alcohol, is classified as a food additive by the European Commission Regulation No 231/2012 and is produced through carbohydrate fermentation. With a very low glycemic index (GI: 0), it is considered safe for consumption by individuals with diabetes and obesity (76). While it has negligible caloric content and minimal side effects, excessive consumption may lead to laxative effects. However, studies have shown that consumption of up to 50 g of erythritol does not induce laxative symptoms (77).

3-Phenyllactic acid is a compound produced by lactic acid bacteria and exhibits antifungal properties (78). To date, there is limited literature on its potential hypocholesterolemic or hypolipidemic effects. Therefore, this bioactive compound may have a weak or even negative contribution to cholesterol reduction and lipolysis inhibition based on the OPLS analysis

Another bioactive component, 3-hydroxy-3-methylglutaric acid (HMG) or meglutol, is an organic acid complex with hypolipidemic effects and is commonly found in seeds (79). Recent research by Iman et al. (80) detected increased levels of this compound in fermented edamame, suggesting a role in regulating blood cholesterol. Subsequent research by the same group (81) demonstrated that meglutol can lower LDL-cholesterol in plasma and is found in the highest concentrations in pea tempe. The mechanism of action involves inhibiting endogenous cholesterol synthesis by acting as an inhibitor of HMG-CoA reductase, the enzyme that converts mevalonate to cholesterol (82). This mechanism is similar to that of statins, which are widely used to treat hypercholesterolemia.

While the mechanism of gamma aminobutyric acid (GABA) in lowering blood cholesterol is less well understood, numerous *in vivo* and clinical studies have shown that GABA supplementation can reduce total cholesterol, LDL-cholesterol, and triglycerides while increasing HDL cholesterol (83–85). GABA has also been shown to prevent atherosclerosis by inhibiting the formation of foam cells in macrophages, thus reducing the risk of blood clots (86). Additionally, GABA can decrease adipogenesis and lipogenesis while increasing energy expenditure. In obese rats, GABA supplementation prevented weight gain despite a high-fat diet (87).

GABA is produced through the decarboxylation of glutamate. It can be synthesized chemically or, more environmentally friendly, through fermentation. During fermentation, lactic acid bacteria produce glutamate decarboxylase (GAD) enzyme, which catalyzes the synthesis of GABA from L-glutamate using pyridoxal-5-phosphate (PLP) as a cofactor (88, 89). Therefore, the fermentation process in tempe production enhances GABA concentration.

Similar to GABA, the soybean isoflavones daidzein and genistein have been reported to have hypocholesterolemic effects, although the mechanisms are not fully understood. Genistein is more potent than daidzein in lowering plasma cholesterol (90, 91). Genistein has the potential to inhibit cholesterol synthesis by targeting HMG-CoA reductase. Computational studies by Hermanto et al. (92) suggest that genistein has a high affinity for HMG-CoA reductase and low toxicity, making it a promising candidate for cholesterol reduction. Isoflavones also exhibit anti-obesity effects, with genistein preventing fat accumulation and daidzein reducing inflammation in obese individuals (93). Isoflavone consumption can prevent adipogenesis through specific mechanisms in the body (94, 95).

## 4 Conclusion

This study demonstrated that tempe flour exhibits higher cholesterol binding activity and lipase inhibition activity compared to soybean flour. Metabolomic analysis using the orthogonal partial least squares (OPLS) method revealed a group of biomarker compounds that were strongly and positively correlated with cholesterol binding and anti-lipase bioactivities. These biomarkers included bioactive compounds derived from amino acids and organic acids. GC–MS analysis identified higher levels of bioactive compounds such as meglutol (3-hydroxy-3-methylglutarate), GABA (gamma aminobutyric acid or 4-aminobutyric acid), and isoflavones in tempe flour. These compounds are believed to play significant roles in human cholesterol and lipid metabolism.

## Data Availability

The original contributions presented in the study are included in the article/Supplementary material, further inquiries can be directed to the corresponding author.
