# Supplementary material for: Metabolite profiling of bioactive compounds in tempe flour and its potential as a hypocholesterolemic functional food
Source: Front Nutr. 2025 Nov 12;12:1622952. doi: 10.3389/fnut.2025.1622952 (PMC12648969; doi:10.3389/fnut.2025.1622952)
Supplement: Supplementary file 1 [file Data_Sheet_1.pdf]

Figure S1 : Y-Related Coefficient (A), S-plot (B), and VIP (C) metabolites correlated with cholesterol binding

A

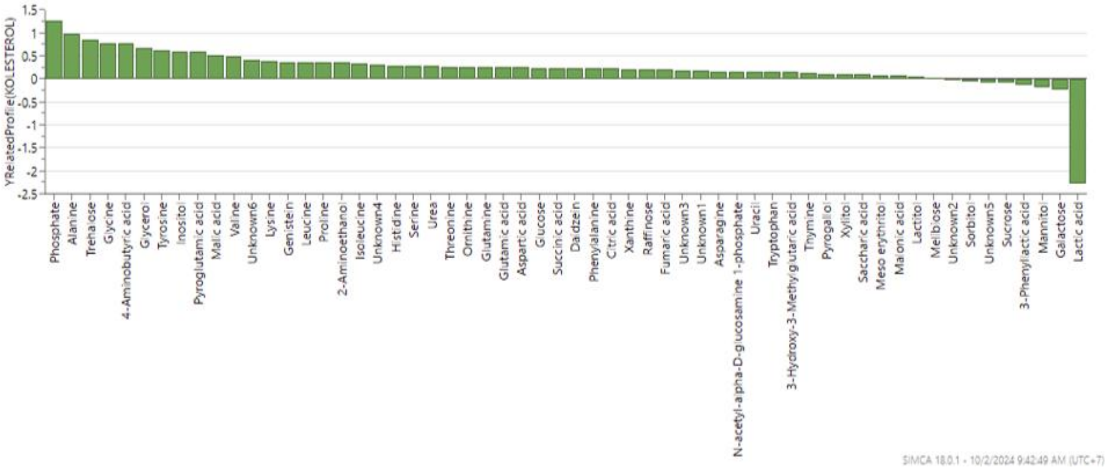

Figure S1A: Metabolite (X axis) and Y-related coefficient (Y axis). Sample from tempe flour (n=4) and soy flour (n=4). Analysis of the correlation between metabolite (GC-MS) and bioactivity cholesterol binding (*Lieberman Burchard*)

Y-related Coefficient Plot from an OPLS-DA Model, Showing the most influential metabolites in differentiating groups based on cholesterol binding activity. The Y-axis shows the Y-related Coefficient, where a positive value indicates a positive correlation with cholesterol binding (i.e., these metabolites are higher in the high-cholesterol binding activity group), while a negative value indicates a negative correlation (higher in the low-cholesterol binding activity group). Metabolites with the largest coefficients (Phosphate, Alanine, Trehalose, etc.) have the greatest impact on the model and are potential biomarkers for differences in cholesterol binding activity

B

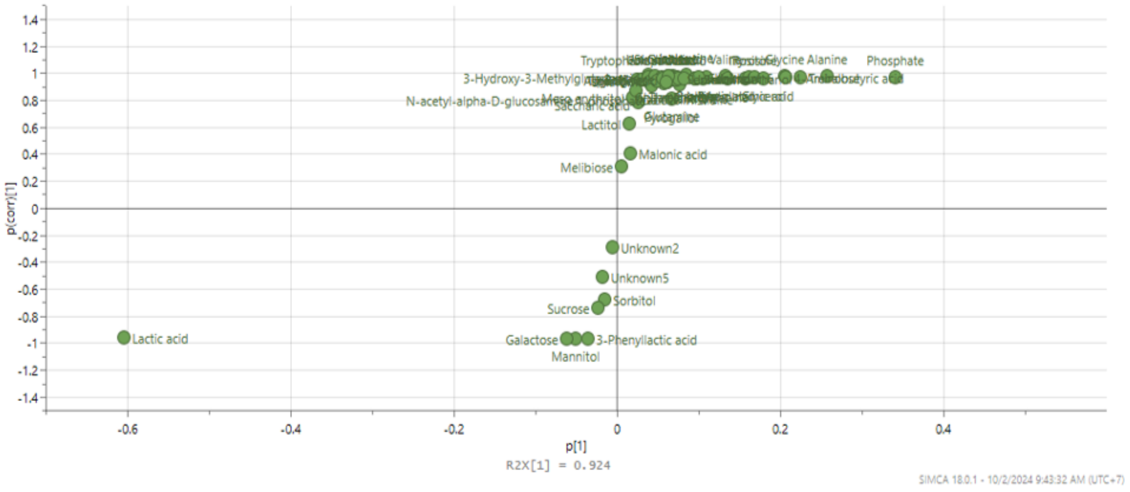

Figure S1B: S-Plot Derived from an OPLS Model highlighting metabolites contributing to group separation (e.g., high vs. low Cholesterol binding activity, n=4 per group) Using GC-MS and bioactivity cholesterol binding (*Lieberman Burchard*)

The X-axis (p[1]) represents the covariance between the metabolites and the predicted Y variable, indicating the magnitude of a metabolite's change. The Y-axis (p(corr)[1]) represents the correlation of each metabolite with the predicted Y variable, indicating the reliability of the change. Metabolites located in the upper right and lower left quadrants are the most influential, possessing both high covariance and strong correlation. For instance, Lactic acid (lower left) shows a strong negative correlation and high contribution, suggesting it is significantly lower in one group, while metabolites like Phosphate (upper right) show a strong positive correlation and high contribution, indicating they are significantly higher in the other group. The R2X[1] value of 0.924 indicates that 92.4% of the variance in the X data (metabolites) is explained by the first predictive component of the model. This plot effectively identifies potential biomarkers differentiating the sample groups.

C

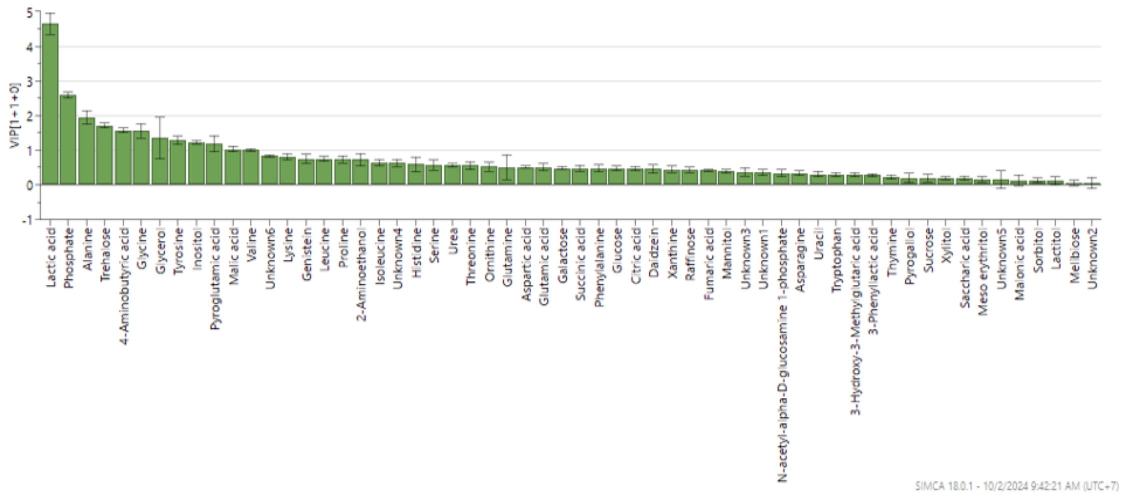

Figure S1C: VIP (Variable Importance in Projection) Plot Derived from an OPLS Model, identifying key metabolites Responsible for differentiating sample Groups (e.g., high vs. low cholesterol binding activity, n=4 per group) Using GC-MS and bioactivity cholesterol binding (*Lieberman Burchard)*

The Y-axis represents the VIP score for each metabolite, which quantifies the overall contribution of that metabolite to the OPLS model's ability to explain and predict the Y variable (e.g., cholesterol binding activity). The X-axis lists the individual metabolites. Metabolites with VIP scores greater than 1 (e.g., Lactic acid, Phosphate, Alanine, Trehalose, and 4-Aminobutyric acid) are considered to be the most influential and statistically significant in discriminating between the sample groups. These metabolites represent strong candidates for biomarkers related to the condition or grouping under study. The error bars indicate the variability of the VIP score estimates.

Figure S2 : Y-Related Coefficient (A), S-plot (B), and VIP (C) metabolites correlated with anti lipase

A

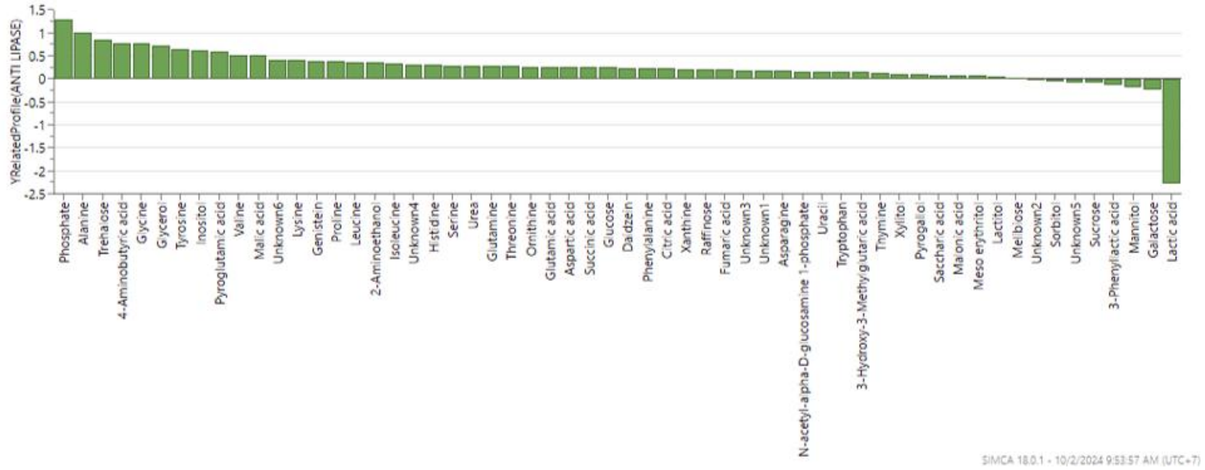

Figure S2A: Y-Related Coefficient Plot Derived from an OPLS Model, illustrating metabolites correlated with Anti-Lipase Activity in soy and tempe flour (n=4 per group) Using GC-MS and anti-lipase analysis.

The Y-axis represents the Y-Related Coefficient, which indicates the strength and direction of the correlation between each metabolite and the anti-lipase activity. Positive coefficients (e.g., Phosphate, Trehalose, 4-Aminobutyric acid) signify that these metabolites are positively associated with anti-lipase activity (i.e., higher levels in samples with stronger anti-lipase effect). Conversely, negative coefficients (e.g., Lactic acid, Galactose, Mannitol) suggest a negative association, meaning they are lower in samples with stronger anti-lipase activity. Metabolites with larger absolute coefficient values have a more significant influence on the anti-lipase effect, serving as potential biomarkers or targets.

B

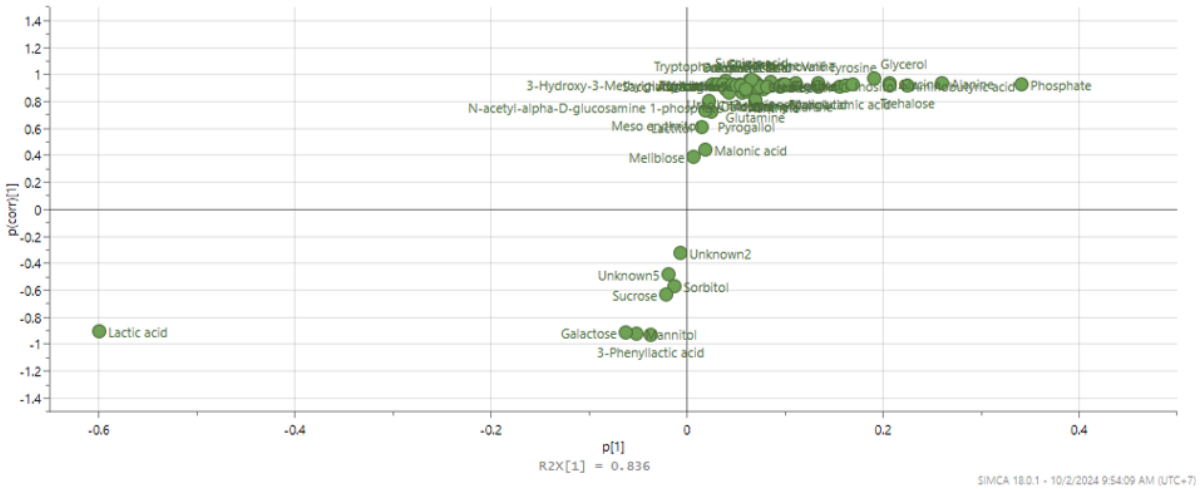

Figure S2B: S-Plot Derived from an OPLS Model, Visualizing Metabolites Contributing to the Differentiation of Sample Groups (e.g., High vs. Low Anti-Lipase Activity, n=4 per group) Using GC-MS and anti lipase analysis.

The X-axis (p[1]) represents the modeled covariance between each metabolite and the Y variable (e.g., anti-lipase activity), indicating the magnitude of the metabolite's change. The Y-axis (p(corr)[1]) represents the modeled correlation of each metabolite with the Y variable, indicating the reliability or significance of that change. Metabolites located at the extreme ends of the plot (upper right and lower left corners) are the most influential, possessing both high covariance and strong correlation with the anti-lipase activity. For instance, Lactic acid (lower left quadrant) shows a strong negative correlation and high contribution, suggesting it is significantly lower in the group with higher anti-lipase activity. Conversely, metabolites in the upper right, such as Phosphate, show a strong positive correlation and high contribution, indicating they are significantly higher in the group with higher anti-lipase activity.

C

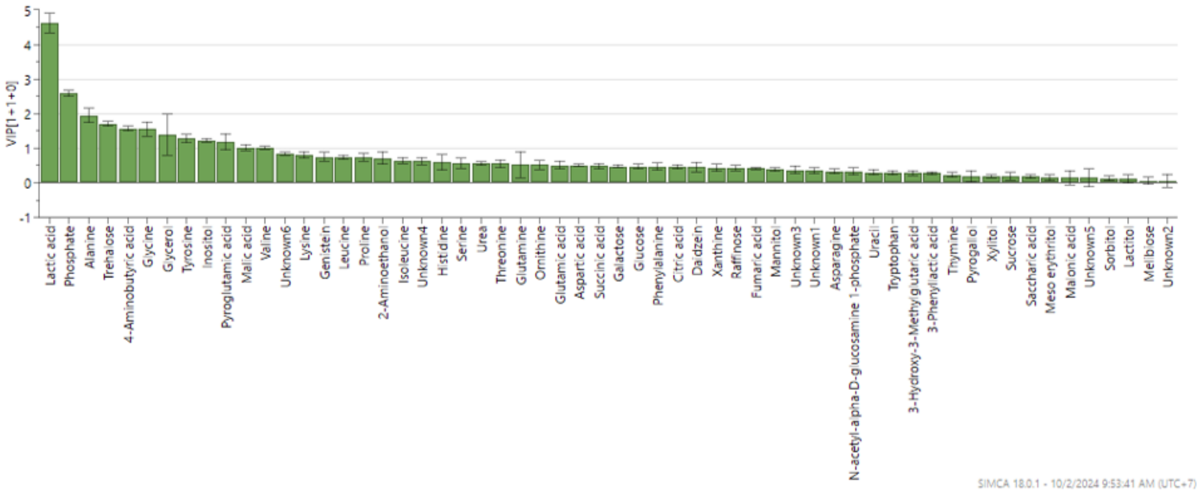

Figure S2C: VIP (Variable Importance in Projection) Plot Derived from an OPLS Model, Identifying key metabolites most responsible for differentiating sample groups (n=4 per group) Using GC-MS and anti lipase analysis.

The Y-axis represents the VIP score for each metabolite, which quantifies the overall contribution of that metabolite to the OPLS model's ability to explain and predict the Y variable (e.g., anti-lipase activity). The X-axis lists the individual metabolites, ordered by their VIP scores. Metabolites with VIP scores greater than 1 (e.g., Lactic acid, Phosphate, Alanine, Trehalose, and 4-Aminobutyric acid) are considered to be the most influential and statistically significant in discriminating between the sample groups. These highly ranked metabolites represent strong candidates for biomarkers or therapeutic targets related to anti-lipase activity. The error bars indicate the variability or standard deviation of the VIP score estimates.

Figure S3 : OPLS permutation plot: cholesterol binding (A) and anti-lipase (B)

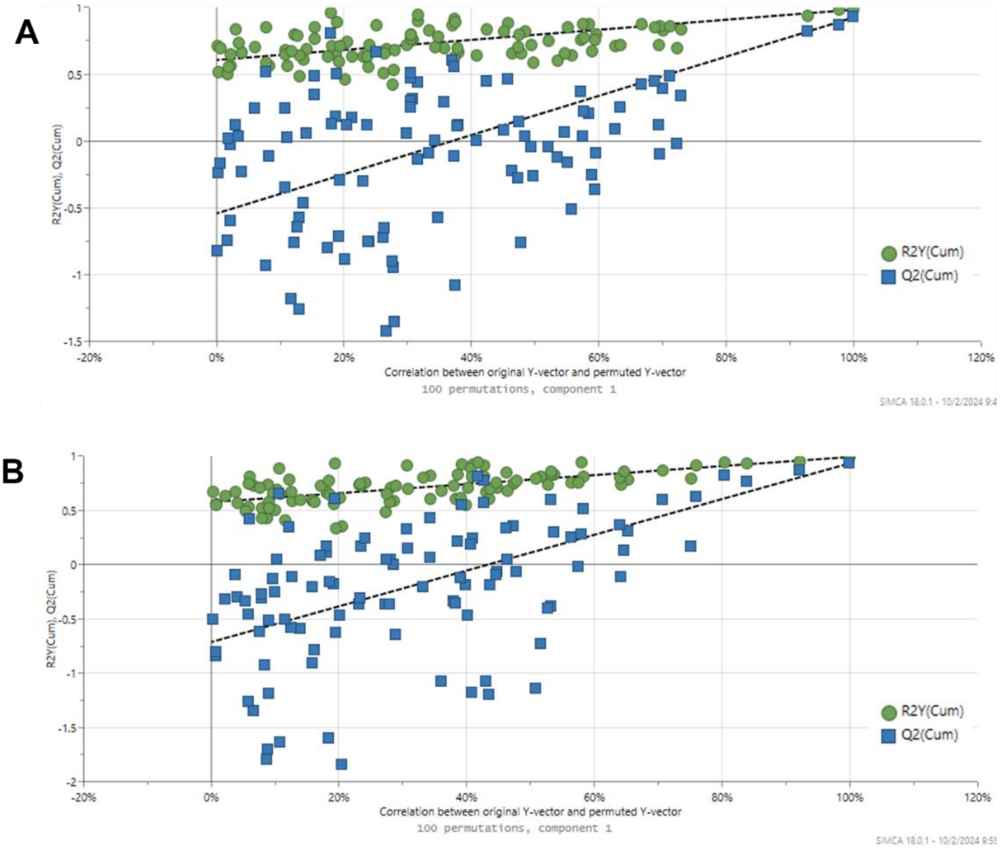

In this study, the two OPLS models underwent 100 randomization runs. Figure A and B clearly illustrates that the performance of both bioactivity models is good, as the permuted R^2Y and Q^2Y values are smaller than the actual R^2Y and Q^2Y values. Visually, the permuted values (represented by the green circles and blue squares) accumulate in the lower-left quadrant, while the actual R^2Y and Q^2Y values are located in the upper-right quadrant.

Table S1 CV Anova Cholesterol Binding Model

M1	SS	DF	MS	F	p	SD
**KOLESTEROL**						
**Total corr.**	0.290996	7	0.041571			0.203889
**Regression**	0.269239	3	0.089746	16.4996	0.010217	0.299577
**Residual**	0.021757	4	0.005439			0.073752

p<0.05 : The model provided exhibits **strong and reliable** performance

Table S2 CV Anova Anti Lipase Model

M1	SS	DF	MS	F	p	SD
**ANTI LIPASE**						
**Total corr.**	0.253994	7	0.036285			0.190486
**Regression**	0.23742	3	0.07914	19.1	0.007808	0.281319
**Residual**	0.016574	4	0.004143			0.06437

p<0.05 : The model provided exhibits **strong and reliable** performance

Model validation using the CV-ANOVA test yielded a p-value of 0.01 for the cholesterol-binding model and 0.007 for the anti-lipase model. A p-value for CV-ANOVA <0.05 indicates a model with good reliability (Eriksson et al., 2008)
